# Sudden cardiac death of Duchenne muscular dystrophy with NT-proBNP in pericardial fluid as a useful biomarker for diagnosis of the cause of death: a case report

**DOI:** 10.1080/20961790.2017.1333249

**Published:** 2017-06-22

**Authors:** Mengzhou Zhang, Rui Zhao, Tianshui Yu, Jiaoyong Li, Miao Zhang, Shukun Jiang, Linlin Wang, Guohua Zhang, Rubo Li, Baoli Zhu, Dawei Guan

**Affiliations:** a Department of Forensic Pathology, China Medical University School of Forensic Medicine, Shenyang, China; b Remote Forensic Consultation Center, Collaborative Innovation Center of Judicial Civilization, China University of Political Science and Law, Beijing, China

**Keywords:** Forensic medicine, NT-proBNP, sudden cardiac death, Duchenne muscular dystrophy, left ventricular dysfunction

## Abstract

Duchenne muscular dystrophy (DMD) is one of the most common and severest muscular dystrophies. Although it can be a cause of death when associated with cardiac muscle and/or respiratory muscles, no cases of sudden deaths in the setting of undiagnosed DMD with cardiac involvement have been reported in the literatures. Previous studies showed that N-terminal-proBNP (NT-proBNP) was a robust laboratory biomarker to diagnose and monitor cardiac failure in clinical situations, suggesting that it may be used as an auxiliary indicator for diagnosis on left ventricular dysfunction in sudden cardiac deaths in forensic settings. Here, we reported a case of 29-year-old man who died suddenly. Autopsy revealed that muscles of the body were almost replaced by fatty and fibrotic tissues. The heart was enlarged with disarray and degeneration of cardiomyocytes in cardiac muscle. Total absence of dystrophin was detected by immunohistochemical staining, which confirmed DMD. Postmortem biochemical test of pericardial fluid revealed a high level of NT-proBNP, indicating dysfunction of the left ventricle before death. The cause of death was certified as an early dilated cardiomyopathy (DCM)/dysfunction of the left ventricle secondary to DMD, suggesting that sudden cardiac death with cardiac dysfunction could be identified by immunohistochemical method in combination with pericardial fluid NT-proBNP determination after systemic autopsy.

## Introduction

Duchenne muscular dystrophy (DMD) is an X-linked recessive disease affecting 1 among 3 600–6 000 living male births [1], characterized by distribution of muscle wasting, progressive loss of skeletal and cardiac muscle function with calf hypertrophy [2]. DMD occurs as a consequence of mutations (mainly deletions) in the dystrophin gene (locus Xp21.2), leading to the total absence of protein dystrophin, which results in muscle necrosis and replacement by adipose and fibrotic tissues. DMD patients, who are usually diagnosed at 3–5 years old, present symptoms such as muscle weakness, inability to stand up without support (Gower's sign), inability to climb stairs, repeated fall, palpitation, forced breathing and fatigue [3]. The mean age of death of DMD patients is around 19 years if proper clinical treatment is not given [2]. The patients, particularly in prolonged course of this disease, are vulnerable to the highest risk of cardiac complications. The leading cause of death is cardio-respiratory failure, followed by pneumonia, multi-organ failure, malnutrition, fat embolism and adrenal insufficiency [4]. Given the diagnosis of DMD relies on clinical history and manifestations, it is a challenge to determine the cause of death in patients with DMD if no clinical information is provided in forensic practice.

Here, we reported a sudden unexpected death of a DMD patient who had not been diagnosed and treated. The cause of death was postulated to be an early dilated cardiomyopathy (DCM)/left ventricular (LV) dysfunction secondary to DMD, which was identified by immunohistochemical method in combination with pericardial fluid N-terminal-proBNP (NT-proBNP) determination after systemic autopsy.

## Case

A 29-year-old Chinese man who had no disease history expired suddenly and fell down from bed at home. As his death was sudden and unexpected, a forensic pathological team was commissioned to conduct a full postmortem examination in order to constitute a reportable death.

### External examination

The decedent was a well-nourished adult male of appearance in keeping with his stated age, measuring 170 cm for a weight of 80 kg (body mass index = 27.7). In addition to peripheral subungual cyanosis, there was no evidence of other pathological findings.

### Forensic autopsy

**Figure 1.**
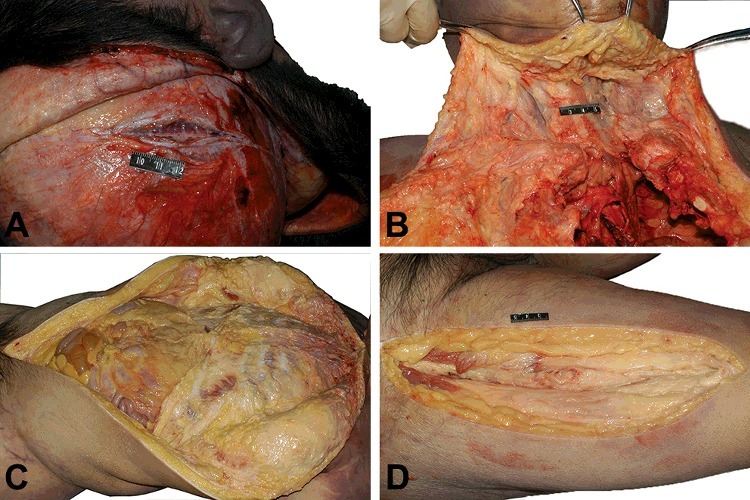
External examination reveals that temporal muscles (A), cervical muscle group (B), abdominal muscles (C) and thigh muscles (D) are almost replaced with adipose tissue.

**Figure 2.**
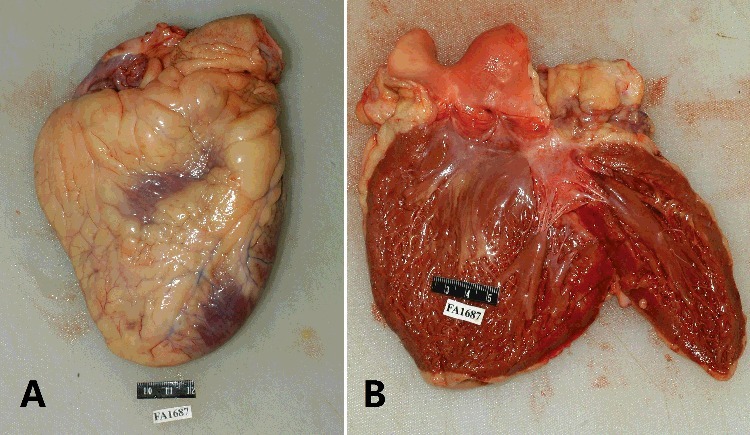
Gross examination shows that the heart is enlarged and the surface of the heart is extensively covered with adipose tissue (A). The left ventricle appears dilated. Scattered hyperplasia of adipose tissue can be seen beneath the endocardium of the left ventricle (B).

Skeletal muscles of the body were almost replaced with adipose tissues, which could not be clearly discriminated (Figure 1). The heart was enlarged and weighed 420 g (the normal weight for men is 300–350 g [5]). The surface of the heart was extensively covered with adipose tissue (Figure 2(A)). A myocardial bridge was found in the intermediate portion of the left anterior descending artery. A focal atherosclerotic plaque with slight luminal stenosis was observed in the right main coronary artery. The left ventricle was dilated, the ventricular chamber diameter was 6.3 cm and scattered hyperplasia of adipose tissue could be seen beneath the endocardium of the left ventricle (Figure 2(B)). The thickness of the left ventricle was 1.2 cm (normal thickness, 1.3–1.5 cm), while the thickness of the right ventricle was 0.1 cm (normal thickness, 0.3–0.5 cm). The cardiac muscle of the right ventricle was abundant with fatty tissues. The lungs were overweight (right: 550 g, left: 500 g), appearing pulmonary oedema. The weights of brain, liver and spleen were 1 430, 1 500 and 240 g, respectively. The weight of the kidneys was 150 g in each. The parenchyma of pancreas, thymus and thyroid gland showed a large content of adipose tissues on cross sections. The macroscopic examinations of other organs showed no obvious abnormalities.

### Histological analysis

**Figure 3.**
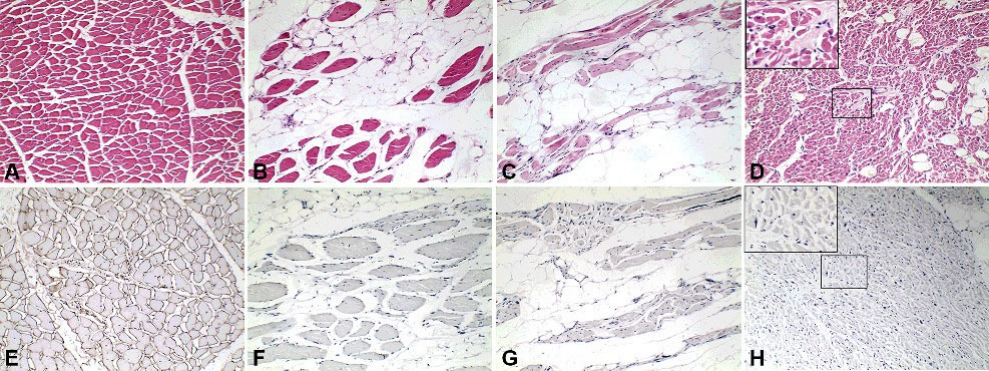
Microscopically most skeletal muscle fibres are replaced with adipose and fibrotic tissues. There are also evidences of marked variability in fibre size, focal fibre necrosis and invasion by monocytes in skeletal muscles taken from quadriceps (B), diaphragm (C) compared with the normal control (A). Cardiac muscle exhibits variation in fibre size with a disarrayed appearance. The ventricular myocardium is infiltrated by diffused fatty foci. Degeneration of cardiac muscles and focal myocardial fibrosis are found (D). Dystrophin is totally absent in the sarcolemma of quadriceps (F), diaphragm (G) and cardiac muscle (H) compared with the normal control (E). Magnification × 10 (A–H); × 20 (insets in D, H).

A histopathological examination was carried out on the muscles, myocardium, brain, lungs, liver, kidneys, spleen, pancreas, thymus and thyroid gland using standard H&E staining. The veins in lungs and other organs appeared congested and dilated. There was no fat embolism and thrombi in the lungs. Significant histological findings were confined to the heart and the muscles of the body. It was found that most skeletal muscle fibres of quadriceps (Figure 3(B)) and diaphragm (Figure 3(C)) were replaced with adipose and fibrotic tissues. There were also evidences of marked variability in fibre size, focal fibre necrosis and invasion by monocytes compared with the normal control (Figure 3(A)). Cardiac muscle exhibited variation in fibre size with a disarrayed appearance. The ventricular myocardium was infiltrated by diffused fatty foci. Degeneration of cardiac muscles and focal myocardial fibrosis were also observed (Figure 3(D)).

Immunohistochemical staining of dystrophin was performed on the muscles and myocardium samples. Immunohistochemistry revealed total absence of dystrophin in the sarcolemma of quadriceps (Figure 3(F)), diaphragm (Figure 3(G)) and cardiac muscle (Figure 3(H)) compared with the normal control (Figure 3(E)).

### Laboratory analyses

Samples of heart blood, urine and gastric contents were submitted for screening analyses by gas chromatography and mass spectrometry. Common drugs of abuse and poisons were negative.

Post-mortem biochemical tests were performed on a sample of pericardial fluid with results as follows (standard levels are given in brackets): NT-proBNP 662.8 pg/mL (<115 pg/mL); CK-MB 4 234 ng/mL (340–2 420 ng/mL); cTnT 1 701 ng/mL (181–3 352 ng/mL). The creatine kinase (CK) of serum was 5 226 ng/mL (<250 ng/mL).

## Discussion

The history of DMD has been exhaustively reviewed [3,6]. While the name of Duchenne has received the eponymous honour, the first description of this progressive muscular dystrophy can be attributed to Meryon in 1830 [3].

The diagnosis of DMD should meet the following criteria [1,2,7]: (1) onset of weakness before 5 years old, (2) male sex, (3) proximal muscle weakness, (4) increased serum CK (5) muscle biopsy and/or DNA analyses consistent with DMD. Although DNA analyses are strictly mandatory for carrier studies, prenatal diagnosis and therapies, they have no significant value for positive biopsy diagnosis of DMD [7], especially in forensic situation. Considering some patients with severe complications have non-specific clinical signs [8,9], the final and golden diagnosis is an absence or defection of dystrophin confirmed by immunohistochemistry [1,7,10].

In our case, the skeletal muscles were necrotized and replaced with adipose and fibrotic tissues. Immunohistochemistry revealed the total absence of dystrophin in myocardium, quadriceps and diaphragm. Biochemical examination showed serum CK level was grossly elevated. The results of all examinations and tests confirmed that the decedent was a DMD patient.

The cause of death of DMD patients was respiratory failure and/or cardiac failure. Because respiratory function was more prone to be affected than cardiac function, pneumonia was the main cause of death of DMD patients in the past, which happened around 19 years old. However, with more attention to respiratory care and various forms of assisted ventilation, individuals affected now can survive into their 30–40 years [2,11]. DCM is a common finding in DMD patients, being present in virtually all patients over 18 years old, which lead to advanced heart failure and premature death and can account for an increasing proportion of morbidity and mortality [11,12].

In our case, the heart was larger than the average with dilated ventricle and abnormal ventricular wall thickness. The focal myocardial fibrosis, diffused fatty infiltration and disarray of partial cardiomyocytes were observed morphologically, demonstrating that the decedent suffered from an early DCM [13,14]. Although the heart failure is the leading cause of death in DMD patients, deaths resulting from pneumonia, multi-organ failure, malnutrition, fat embolism and adrenal insufficiency cannot be ignored [4]. It is difficult to make sure the immediate cause of death without any clinical information.

Natriuretic peptides are a group of hormones produced mainly by cardiomyocytes and their productions are up-regulated by increased wall pressure, ischemia or infarction [15]. Cardiac myocytes produce BNP prohormone, proBNP, which is split into NT-proBNP and BNP by the protease furin [16]. After secretion, BNP binds to natriuretic peptide receptors A and C to exert biological effects, including diuresis, vasodilatation and inhibition of renin and aldosterone production. Those characteristics constitute the basis for using NT-proBNP and BNP as conventional biochemical markers of cardiac disease in clinical and many medico-legal situations [16]. BNP has been used to evaluate cardiac dysfunction/DCM in clinical diagnosis of DMD [17,18]. Misumi et al. reported a DMD patient died of LV failure with a markedly increased plasma BNP level [19]. The level of BNP in pericardial fluid is higher than in plasma [20] and can be served as a more sensitive and accurate indicator of LV dysfunction [21,22]. NT-proBNP, which has a longer half-life and greater stability than BNP [15,17], is considered to be a robust postmortem biomarker to diagnose cardiac failure [15,21,23]. What is more, postmortem measurements of NT-proBNP are reliable and compatible with clinical findings [15]. van Bockel et al. showed that the combination of echocardiography and NT-proBNP achieved similar results in the evaluation of LV function in comparison with multigated cardiac radionuclide ventriculography (MUGA) and advised to add NT-proBNP to cardiac assessment of DMD patients [24]. Owing to a high sensitivity for DCM, serum NT-proBNP measurement might be considered as a routine screening and diagnosis of cardiomyopathy in DMD patients [17,24].

In our case, high-level NT-proBNP in pericardial fluid indicated antemortem LV dysfunction and an evidence of an early DCM. The cause of sudden death, considering the involvement of the myocardial fibrosis and infiltration of adipose tissue, should be attributed to ventricular arrhythmias [14,25] and/or acute heart failure [19]. Because no acute myocardial ischemia was found in the heart, the myocardial bridge and coronary atheroma might play a less role in the death of the decedent. The disarrayed appearance and infiltration of adipose tissue in cardiac muscle could not be explained as a result of ischemic insults, which was associated with dystrophin deficiency.

According to the gross and histopathological examination, we excluded the other possible causes of death, such as pneumonia, fat embolism, malnutrition and multiple organ failure. Adrenal insufficiency could also be ruled out because it often occurs in the patients who rely on regular corticosteroids [4]. As mentioned above, it is concluded that the sudden death of the decedent was linked to an early DCM/LV dysfunction secondary to DMD, leading to acute cardiac failure.

To our knowledge, the deaths of DMD patients reported in the literatures all occurred during or following medical management. This is the first case report of sudden unexpected death of undiagnosed DMD, in which NT-proBNP of pericardial fluid was employed as an auxiliary candidate for diagnosis of cause of death in DMD patient. Our present study has some limitations since there was only one case. The applicability and validity of NT-proBNP of pericardial fluid in certifying sudden cardiac deaths should be prospectively investigated in a large number of subjects.
